# Impact of the 13-Valent Conjugated Pneumococcal Vaccine on the Direct Costs of Invasive Pneumococcal Disease Requiring Hospital Admission in Children Aged < 5 Years: A Prospective Study

**DOI:** 10.3390/vaccines8030387

**Published:** 2020-07-15

**Authors:** Sergi Hernández, Encarna Navas, Ignacio Aznar-Lou, Pilar Ciruela, Juan José García-García, Fernando Moraga-Llop, Carmen Muñoz-Almagro, Gemma Codina, Mariona F. de Sevilla, Sebastià González-Peris, Cristina Esteva, Ana María Planes, Conchita Izquierdo, Johanna Martínez-Osorio, Magda Campins, Sonia Uriona, Luis Salleras, Antoni Serrano-Blanco, Mireia Jané, Ángela Domínguez

**Affiliations:** 1 Public Health Agency of Catalonia, Generalitat de Catalunya, 08005 Barcelona, Spain; e.navas@gencat.cat (E.N.); pilar.ciruela@gencat.cat (P.C.); mireia.jane@gencat.cat (M.J.); conchitaizquierdog@gmail.com (C.I.); 2 Teaching, Research & Innovation Unit, Institut de Recerca Sant Joan de Déu, Sant Boi de Llobregat, 08830 Barcelona, Spain; i.aznar@pssjd.org; 3 CIBER de Epidemiología y Salud Pública (CIBERESP), Instituto de Salud Carlos III, 28029 Madrid, Spain; juanjo@sjdhospitalbarcelona.org (J.J.G.-G.); cma@sjdhospitalbarcelona.org (C.M.-A.); cesteva@sjdhospitalbarcelona.org (C.E.); salleras@ub.edu (L.S.); aserrano@pssjd.org (A.S.-B.); angela.dominguez@ub.edu (Á.D.); 4 Hospital Sant Joan de Déu Barcelona, Universitat de Barcelona, Esplugues de Llobregat, 08950 Barcelona, Spain; mariafernandez@sjdhospitalbarcelona.org (M.F.d.S.); jmmartinez@sjdhospitalbarcelona.org (J.M.-O.); 5 Malalties Prevenibles amb Vacunes, Institut de Recerca Sant Joan de Déu, Esplugues de Llobregat, 08950 Barcelona, Spain; 6 Hospital Universitari Vall d’Hebron, 08035 Barcelona, Spain; fmoraga@acmcb.es (F.M.-L.); mgcodina@vhebron.net (G.C.); sgonzalezperis@gmail.com (S.G.-P.); amplanes@gmail.com (A.M.P.); mcampins@vhebron.net (M.C.); urionasonia@gmail.com (S.U.); 7 Departament de Medicina, Universitat Internacional de Catalunya, 08017 Barcelona, Spain; 8 Epidemiology and Public Health Research Group, Vall d’Hebron Institut de Recerca, 08035 Barcelona, Spain; 9 Departament de Medicina, Universitat de Barcelona, 08036 Barcelona, Spain; 10 Parc Sanitari Sant Joan de Déu, Sant Boi de Llobregat, 08830 Barcelona, Spain

**Keywords:** PCV7, PCV13, invasive pneumococcal disease, direct cost

## Abstract

The lack of invasive pneumococcal disease (IPD) cost studies may underestimate the effect of pneumococcal polysaccharide conjugated vaccines (PCV). The objective of this study was to estimate the direct costs of hospitalized IPD cases. A prospective study was made in children aged <5 years diagnosed with IPD in two high-tech hospitals in Catalonia (Spain) between 2007–2009 (PCV7 period) and 2012–2015 (PCV13 period). Costs were calculated according to 2014 Catalan Health Service rates using diagnostic-related groups. In total, 319 and 154 cases were collected, respectively. Pneumonia had the highest cost (65.7% and 62.0%, respectively), followed by meningitis (25.8% and 26.1%, respectively). During 2007–2015, the costs associated with PCV7 serotypes (Pearson coefficient (Pc) = −0.79; *p* = 0.036) and additional PCV13 serotypes (Pc = −0.75; *p* = 0.05) decreased, but those of other serotypes did not (Pc = 0.23 *p* = 0.62). The total mean cost of IPD increased in the PCV13 period by 31.4% (€3016.1 vs. €3963.9), mainly due to ICU stay (77.4%; €1051.4 vs. €1865.6). During the PCV13 period, direct IPD costs decreased due to a reduction in the number of cases, but cases were more severe and had a higher mean cost. During 2015, IPD costs increased due to an increase in the costs associated with non-PCV13 serotypes and serotype 3 and this requires further investigation.

## 1. Introduction

Invasive pneumococcal disease (IPD) is a major cause of morbidity and mortality in children aged < 5 years and occurs mostly as pneumonia or other serious forms such as meningitis, bacteremia or osteoarticular infections. In 2000, 14.5 million cases of IPD were estimated to have occurred in children aged < 5 years, causing 7,350,000 deaths [1]. Subsequently, following the introduction of pneumococcal polysaccharide conjugated vaccines (PCV) in 2015, 3.7 million episodes of IPD were estimated in this age group, causing 294,000 deaths [2]. Despite this reduction, deaths attributable to *Streptococcus pneumoniae* in 2015 accounted for 11% of deaths in children aged < 5 years not infected with HIV. Incidence and mortality rates are higher in developing countries and most deaths occur in the African and Asian regions [3]. The European Medicines Agency (EMA) licensed the seven-valent PCV (PCV7) in February 2001 [4] and, in April and December 2009, the 10-valent PCV (PCV10) [5] and the 13-valent PCV (PCV13) [6], respectively. During 2017, the incidence rate of IPD in Europe was 14.5 cases per 100,000 persons in children aged < 1 year, and 24% of cases in children aged < 5 years were due to PCV13 serotypes [7].

Studies show that, after the introduction of PCV10 and PCV13, there was a decrease in IPD incidence in children and adults [8,9,10,11,12,13,14,15]. However, some reports have observed an increase in IPD in children aged < 5 years due to non-PCV13 serotypes [16].

While the licensing of PCVs has led to numerous changes in the epidemiology of IPD, which have been extensively studied microbiologically and epidemiologically, there are few studies of the costs of IPD [17], which may imply an underestimate of the disease itself and, as a consequence, of the effect of these vaccines.

The costs of IPD vary greatly depending on the geographical scope and design of the study. Each region has its own health policies, its own health system, and its epidemiologic reality in terms of circulating serotypes [18]. This makes it difficult to extrapolate cost studies from one region to another. Likewise, many cost studies only refer to cases of invasive pneumonia in children [19] or are included in studies covering the total cost of community-acquired pneumonia in children and adults [20,21]. Other cost studies that reflect the total clinical manifestations of IPD focus only on adults [22,23].

The objective of this study was to estimate the costs of IPD cases requiring hospital admission in children aged < 5 years in high-tech pediatric hospitals in Catalonia (Spain) following the licensing of the PCV7 and PCV13 and to compare and identify the differences between the two periods and the main factors that may involve variations in the costs of IPD, both globally and in the mean cost per case.

## 2. Materials and Methods

### 2.1. Study Design

A prospective study was conducted in children aged < 5 years diagnosed with IPD in two periods. The first period was between 1 January 2007 and 31 December 2009 and corresponded to the period in which the vaccine licensed was PCV7. The second, from 1 January 2012 to 31 December 2015, corresponded to the period in which the vaccine licensed was PCV13. In both periods, two high-tech pediatric hospitals in Catalonia participated, namely the Sant Joan de Déu Hospital and the Vall d’Hebron Mother-Child Hospital, which account for 31.9% of hospital discharges in Catalonia of children aged < 5 years during the first period and 28.9% during the second period, according to data provided by the Minimum Basic Data Set of Hospital Discharges [24]. The present study was conducted as a secondary analysis and is based on the data of two prospective studies performed by the same research group so all cases included were collected prospectively and consecutively during the two periods.

### 2.2. Case Selection

Patients aged < 5 years hospitalized with a diagnosis of IPD during the study periods in the two participating centers were recruited. IPD was defined as clinical findings of infection together with isolation and/or detection of DNA of the neumolisin gene (ply) and a wzg capsular gene (cpsA) of *S. pneumoniae* by real-time PCR in a normally sterile sample according to the methodology described [25,26].

The strains of *S. pneumoniae* isolated by culture were serotyped using the Quellung reaction or dot blot by the National Center for Microbiology, Majadahonda, Madrid [27]. Identification of the *S. pneumoniae* serotype in samples with negative culture and positive PCR was made by PCR in accordance with the previously reported methodology [25,28].

### 2.3. Demographic, Clinical and Epidemiological Variables

The following demographic, clinical and epidemiological variables were recorded for each case: age, sex, date of birth, date of symptom onset, previous care in other hospitals, hospitalization date, clinical form of IPD (meningitis, septic shock, pneumonia, complicated pneumonia, osteoarticular infection, occult bacteremia and others), complications, intensive care unit (ICU) admission and length of stay, and pre- and post-admission antibiotic therapy (days of treatment and antibiotics administered).

### 2.4. Statistical Analysis

Differences between the two study periods in the demographic, clinical, and epidemiological variables were analyzed using Pearson’s chi-square test for categorical variables and the Student’s t-test for continuous variables. The annual evolution of the costs associated with IPD was analyzed by simple linear regression. The linear relationship was checked by ANOVA tests, and Pearson’s correlation coefficients (Pc) were obtained. The 95% confidence intervals (CIs) were calculated, and values of *p* < 0.05 were considered statistically significant. The analyses were conducted using the Statistical Package for Social Sciences (SPSS 19.0).

### 2.5. Economic Analysis

The costs of IPD cases were calculated taking into account the following health service resource use: hospitalization (differentiating between hospitalization in acute hospital, ICU stay and hospitalization in high-tech hospital), interhospital transfer, follow-up medical visits, and outpatient treatment with antibiotics. The unit costs of each resource were the rates published by the Catalan Health Service for the provision of health services through the network of public hospitals and other suppliers.

The cost of hospitalization was estimated using the diagnostic-related group (DRG) methodology. This method is based on two concepts: the relative resource intensity (RRI), which takes into account the complexity of the cases attended, and the intensity relative hospital intensity (RHI), which measures the structure that hospital has to address the disease. Both concepts are regulated annually by the Catalan Health Service [29].

The mean cost per hospital discharge for each facility was calculated using the following formula [30].

*Mean Hospital Discharge Cost* = *65*% *RHI* + *35*% *RRI* (1)

Sant Joan de Déu and Vall d’Hebron are classified as high technology hospitals (Group 4). The mean costs per hospital discharge and the number of cases treated in each hospital were considered, and the weighted average was obtained so that the same unit cost was allocated to the hospital discharge regardless of the period in which the patient entered. The mean costs per hospital discharge related to prior hospitalization were calculated using their own RHI and RRI values.

The 2014 RRI and RHI indices were applied, and costs were expressed as 2014 euros [30]. Days of acute hospitalization were differentiated from those of ICU hospitalization. The cost per hospital discharge included all diagnostic tests and pharmacological treatments required during hospitalization. The days of ICU stay were added to this cost.

The unit cost of follow-up visits after hospital discharge was considered according to the structural complexity of the hospital center [30], while the unit cost of a primary care center visit was the same for all Catalonia [31].

To establish the unit cost of inter-hospital transfer, we differentiated between scheduled and urgent services, defined as those that accounted for direct entry into the hospital ICU. Since the amount of an urgent inter-hospital transfer is not published in the official documents, it was estimated to be twice the amount of a non-urgent transfer [32,33].

The unit cost of administering outpatient antibiotics was calculated from the public sale price of the prescribed generic antibiotic and only the portion financed by the Catalan Health Service was charged as a cost.

### 2.6. Data Confidentiality and Ethical Aspects

No diagnostic tests were made or samples taken from any participant in addition to those required by routine care. The study complies with the principles of the Declaration of Helsinki and the legal structure according to international human rights and biomedicine and personal data protection legislation.

The Ethics Committee of Hospital Sant Joan de Déu approved the study (CEIC PIC-52-11; approved on 14/11/2011). Informed consent signed by parents or legal guardians was given for all participants. All data were treated as confidential and records were accessed anonymously.

## 3. Results

During the two study periods, 473 cases were collected: 319 (67.4%) during the first period (88 in 2007, 98 in 2008, and 133 in 2009) and 154 (32.6%) during the second (45 in 2012, 34 in 2013, 35 in 2014, and 40 in 2015). The first half of Table 1 shows the clinical and sociodemographic characteristics of patients included: 56.4% (267) of the cases were male and 43.6% (206) female. Patients aged < 1 year accounted for 16.3% of cases (77 cases), patients aged 12–23 months for 26.8% (127 cases), and patients aged 24–59 months 56.9% (269 cases). In total, 303 (64.1%) cases were treated at Sant Joan de Déu and 170 (35.9%) at Vall d’Hebron. In both study periods, pneumonia was the most common clinical presentation (79.6% and 67.5%, respectively) followed by meningitis (9.1% and 11.7%, respectively). The highest percentage of meningitis cases were in 2013 and 2015, 20.6% and 15.0%, respectively (annual data provided on demand).

Of the 319 IPD cases in the first period, 300 (94.0%) were serotyped. Of these, 27 (9%) were PCV7 serotypes, 182 (60.7%) were serotypes added to the PCV13, and 91 (30.3%) were non-PCV13 serotypes. During the second period, 149 (96.7%) cases were serotyped; 19 (12.7%) were PCV7 serotypes, 70 (47.0%) were serotypes added to the PCV13, and 60 (40.3%) were non-PCV13 serotypes.

### 3.1. Health Resources Associated with IPD

The second half of Table 1 shows the health resources associated with IPD in the study periods. During the second study period, there was a higher proportion of patient transfers from their hospital of origin to one of the two study hospitals (24.1% vs. 33.8%; *p* = 0.028), especially for meningitis (24.1% vs. 55.6%; *p* = 0.029). The proportion of urgent transfers was higher during the second study period both overall (14.3% vs. 38.5%; *p* = 0.002), and for pneumonia (4.4% vs. 22.2%; *p* = 0.008) (data on clinical entity provided on demand).

More cases were admitted to the ICU during the second study period overall (13.8% vs. 24.0%; *p* = 0.006) and for cases of pneumonia (5.5% vs. 16.3%; *p* = 0.001). The mean number of ICU days per patient during the first period was 0.8 days and peaked in 2009 (1.2 days). In 2007 and 2008, the mean ICU stay per patient was 0.5 days. During the second period, the mean number of ICU days per patient was 1.6. The mean days of ICU stay in 2012, 2013, 2014, and 2015 were 1.3, 2.1, 0.8, and 2.4 days, respectively.

No significant differences were found between the two study periods in the mean duration of hospitalization and outpatient treatment according to the clinical presentation, except for meningitis, where the days of hospital antibiotic treatment were greater during the second study period (14.5 days vs. 22.6 days, *p* = 0.018). There were no differences in hospital and primary care follow-up visits.

### 3.2. Distribution of the Cost of IPD According to Clinical form and Health Resources

During the two study periods, pneumonia was the clinical presentation with the highest cost (65.7% and 62.0%, respectively), followed by meningitis (25.8% and 26.1%, respectively), other forms of IPD (5.7% and 7.4%, respectively), and non-focal bacteremia (2.8% and 4.4%, respectively).

**Figure 1:**
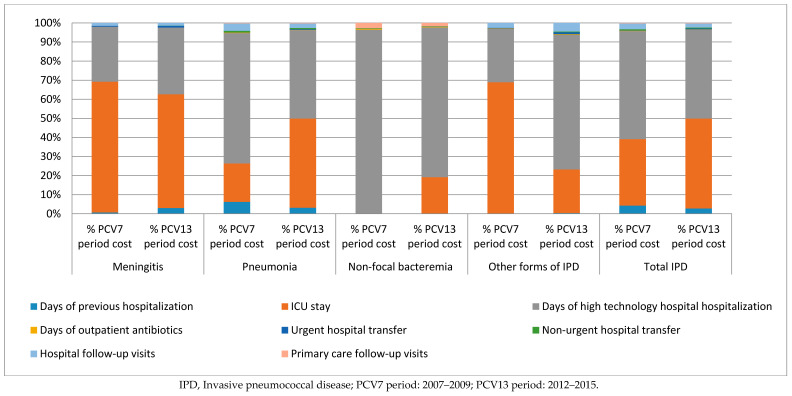
Total cost of IPD according to clinical presentation and study period.

During the first period, the greatest overall costs were due to hospitalization in study hospitals (56.6%), days of ICU admission (34.9%), and days of previous hospitalization (4.3%). During the second study period, the greatest overall costs were days of ICU admission (47.1%), hospitalization in study hospitals (46.6%), and days of previous hospitalization (2.8%) (Figure 1).

For meningitis, in both study periods, the ICU stay generated the most costs (68.5% and 59.6%, respectively) followed by hospitalization in study hospitals (28.8% and 35.0%, respectively), and hospital follow-up visits (1.5%) during the first study period, and days of previous hospitalization (3%) in the second period.

For pneumonia, the ICU stay went from second to first position as the service that generated the most costs (20.2% during the first study period and 46.7% during the second).

For non-focal bacteremia, days of hospitalization (96.4%) were the costliest service during the first study period, followed by primary care follow-up visits (2.8%) and days of outpatient antibiotic treatment (0.5%). During the second study period, days of hospitalization (78.4%) were the costliest service, days of ICU stay (19.2%) occupied the second place, followed by primary care follow-up visits (1.8%).

### 3.3. Annual Evolution of the Costs Associated with IPD

The evolution of the total annual costs associated with IPD during the two study periods is shown in Table 2. The direct costs associated with IPD peaked in 2009. The highest per-patient costs were recorded in 2013 and 2015.

**Figure 2:**
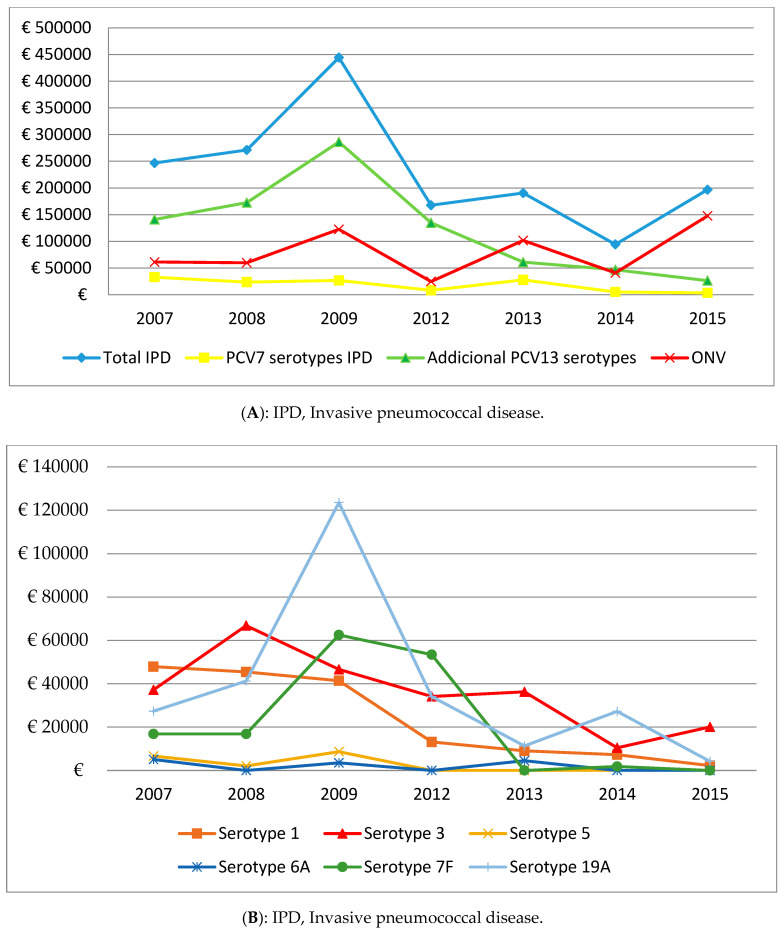
(**A**) Annual evolution of costs associated with IPD according to PCV serotypes; and (**B**) Annual evolution of costs associated with IPD according to PCV serotype.

Depending on the health resources used, there was a decrease in all costs, except for urgent hospital transfers and the ICU stay. The decrease was significant in the costs related to prior hospitalization (Pc = −0.95; *p* = 0.005), hospitalization in study hospitals (Pc = −0.90; *p* = 0.006), days of outpatient antibiotic treatment (Pc = −0.81; *p* = 0.026), non-urgent hospital transfers (Pc = −0.92; *p* = 0.003), and hospital follow-up visits (Pc = −0.84; *p* = 0.017). There was a significant decrease in the direct costs associated with PCV7 serotypes (Pc = −0.79; *p* = 0.036) but not in those associated with the serotypes added to the PCV13 (Pc = −0.75; *p* = 0.05) or in the total annual direct costs of IPD (Pc = −0.63; *p* = 0.131). The direct costs associated with non-PCV13 serotypes increased (Pc = 0.23; *p* = 0.625) (Figure 2A,B).

### 3.4. Variation in the Mean Cost of IPD

The total mean cost of IPD increased in the second study period by 31.4% (€3016.1 vs. €3963.9) (Table 3). The greatest increase in costs was for non-focal bacteremia (57.3%), followed by pneumonia (46.3%) and meningitis (3.2%). The costs of other focal IPDs fell by a mean of −42.8%.

The greatest increases in costs were for urgent intra-hospital transfers (276.6%) and days of ICU stay (77.4%), due to the increase in these resources in treating pneumonia (551.3% and 238.9%, respectively). The costs of days of hospital stay in study hospitals increased by 8.1%, while those of hospitalization in pre-transfer hospitals decreased by 13.9% (Table 3).

### 3.5. Variation in the Proportion of Costs Associated with Serotypes

**Figure 3:**
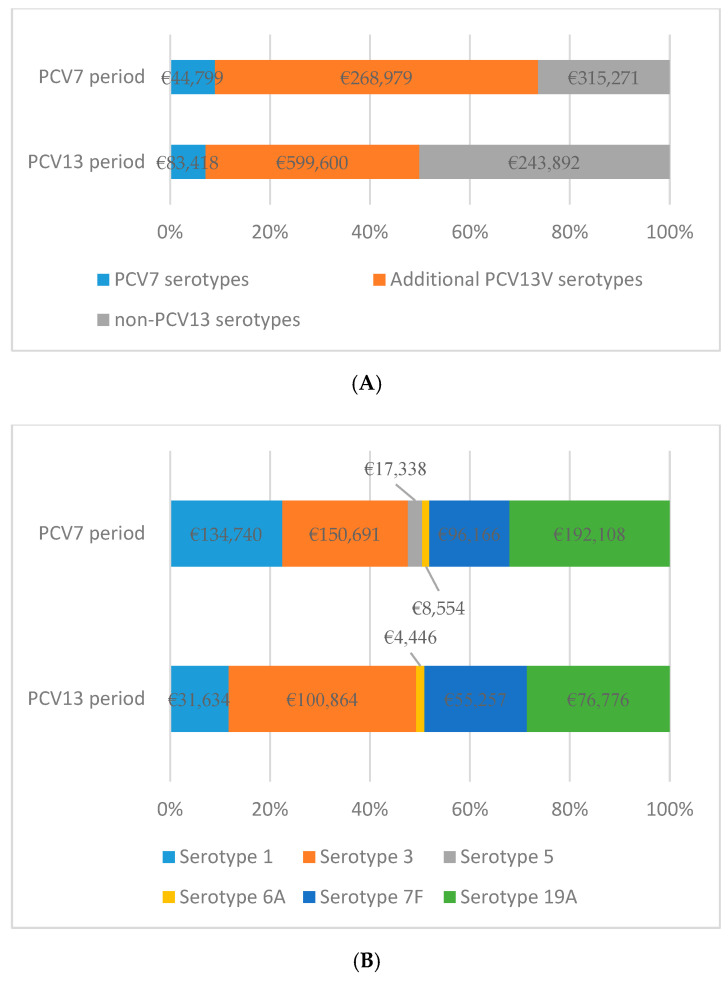
(**A**) Variation in the proportion of IPD costs according with PCV serotypes in the two study periods; and (**B**) variation in the proportion of IPD costs according with additional PCV13 serotypes in the two study periods.

During the two study periods, the proportion of IPD costs caused by PCV7 serotypes decreased slightly (9.0%, and 7.1%, respectively), while there was an important decrease in the proportion of the costs associated with serotypes added to the PCV13 between the two periods (64.7% and 42.8%, respectively). The proportion of the cost of IPD caused by non-PCV13 serotypes increased in the second period (26.3% and 50.1%, respectively) (Figure 3A).

Changes in the proportion of costs associated with serotypes added to the PCV13 were observed between the two study periods. In the first period, the serotypes that produced the highest costs were 19A and 3 (32.0% and 25.1% of the total cost, respectively) followed by serotype 1 (22.5% of the total cost). In the second period, serotype 3 accounted for 37.5% of the total costs associated with serotypes added to the PCV13 followed by serotype 19A (28.5%) and serotype 7F (20.5%) (Figure 3B).

## 4. Discussion

Invasive pneumococcal disease entails a high cost for the health system. Our results show that, after the introduction of PCV13, the direct costs associated with IPD in children aged < 5 years in Catalonia (Spain) decreased globally and for all clinical presentations due to a reduction in the number of cases. These results are comparable to the decrease in hospitalizations observed by Baldo et al. [20] in children aged 0–4 years in the Venetto area. In both study periods, pneumonia was the costliest clinical presentation, followed by meningitis. However, meningitis had the highest mean cost, followed by pneumonia. The years 2013 and 2015 had the highest proportion of patients with a clinical presentation of meningitis and, therefore, the years with the highest cost per patient. This is consistent with the findings of Ceyhan et al. [34] and is due to the fact that meningitis is a more severe disease involving greater use of health resources. The fact that more than 60% of the costs of IPD in the two study periods were associated with pneumonia means that preventing this presentation alone would affect much of the cost associated with IPD. Hernandez et al. [35] found that an increase in pneumococcal pneumonia was associated with children aged 2–4 years in epidemic influenza periods. Consideration should be given to influenza vaccination in this age group, as the double objective of preventing influenza infection and the increase in associated cases of pneumococcal pneumonia would be achieved.

The health resources that generated the most costs varied from one study period to the other. During the first period, the days of hospitalization in study hospitals generated the highest costs. Lagos et al. [36] calculated that 70–75% of the total costs of IPD were due to days of hospitalization. During the second period, the health service that generated the highest costs was the ICU stay. This increase was particularly relevant for pneumonia, where not only the costs of the ICU stay increased but also those of urgent transfers. This, together with the small variations in the costs of the other study items implies greater severity in this type of clinical form. Brotons et al. [19] identified the ICU stay and complications during hospitalizations as factors that increased the cost of the clinical form of pneumonia. We found that the ICU stay was the factor that most increased the cost per IPD patient overall. The years that had the highest number of ICU days per patient also had the highest overall cost per patient.

The use of major health resources increased during the second study period, resulting in increased mean costs in general, as well as for meningitis, pneumonia, and non-focal bacteremia. Comparison of the two study periods showed that the factors that increased the most in the second period were the days of ICU admission and urgent health transfers, indicating that the severity of IPD, and therefore the costs, increased during the second study period. In addition, while the overall costs of IPD decreased between the first and second periods due to the reduction in the number of cases, the cases that occurred in the second period were more severe, consumed more health resources, and had a 31.4% higher mean cost. Although some authors had already warned of a possible increase in the severity of IPD after the introduction of PCV [37], to the best of our knowledge, this is the first study in which this increase in severity has been quantified and reflected as an increase in costs.

Brotons et al. [19] and Song et al. [22] found that the time from symptom onset to hospital admission was not a determining factor in establishing the case severity and the consequent increase in costs. The increased severity of IPD cases during the second study period may be related to changes in the circulating serotypes following the introduction of the PCV13 [38]. In the PCV13 period, the direct cost associated with IPD in children aged < 5 years decreased significantly in IPD cases produced by PCV7 serotypes and, above all, in the serotypes added to the PCV13, but not from non-PCV13 serotypes. The increase in non-PCV serotypes cases, together with the reduction in PCV13 serotypes associated with pneumonia (especially serotype 1) could also explain the increase in the diversity of the clinical presentations of IPD and the decrease in pneumonia cases during the second study period. The proportion of costs associated with the six serotypes added to the PCV13 during the second period remained much higher than that of the seven PCV7 serotypes. This might be explained by the licensing of the PCV7 in 2001, resulting in these serotypes being influenced for longer by the effects of the vaccine coupled with the replacement phenomenon, especially that produced by serotypes 1, 3, and 19A [39,40], which occurred, in our study, in 2009. In addition, the distribution of costs associated with IPD produced by the serotypes added to the PCV13 was very uneven. While the proportion of costs associated with serotype 1 decreased, the proportion associated with serotype 3, which is associated with complicated pneumonia [41], rose from 25.1% of the total costs of additional serotypes to 37.5%, and those of serotype 19A remained stable. This may be explained by the ineffectiveness of the vaccine against serotype 3 [42] and the number of vaccine failures recorded for serotypes 3 and 19A [43]. The increase in IPD costs during 2015, with no decrease in vaccine coverage [35], may be a turning point in the downward trend seen since the licensing of PCV13. The increase in costs could be explained by the increase in the costs associated with non-PCV13 serotypes and the serotype 3, the costs of which appear to have increased by the same proportion, as shown in Figure 2A,B. This change in the trend, together with the increased severity of cases, means that IPD surveillance should continue and new vaccines that include serotypes whose choice is based not only on their prevalence but also on the disease severity they cause, are necessary.

Possible confounding variables were taken into account in the interpretation of the results. Regarding the medical environment, in both study periods, the same hospitals participated; both centers were reference hospitals, a similar account of hospital discharges has been maintained, and medical care circuits did not vary and neither were other pediatric hospitals in the region growing or contracting during the study periods. The same research group participated in both study periods and there were no changes in the management of IPD pediatric patients. As our study was carried out only in high-tech hospitals, there may have been a bias to a higher cost of IPD, since the cost of hospitalization is higher than in other hospitals due to the resources available in these centers and there could have been a selection of the most serious cases of IPD. However, the costs associated with IPD in the form of pneumonia found (€3639.9) were similar to the €3909 found by Baldo et al. in a very similar region [20]. The possible selection of the most serious cases of IPD could also result in a selection of some IPD serotypes, but the evolution of the PCV7 serotypes, additional PCV13 serotypes, and non-PCV serotypes found in our study are similar to those of Catalonia as a whole [38]. PCV7 and PCV13 had the same price and in both study periods the vaccine was not included in the recommended schedule of the National Health System and, therefore, parents had to pay for it.

A limitation of the study is that, to determine the impact of PCV13 on the total direct costs of pneumococcal disease, we had no information on outpatient cases which, although having much lower unit costs, have a high prevalence that implies a large burden on the health system. However, the USA study by Huang et al. [44] found that pneumonia represented 22% of all cases but accounted for 72% of health system use, while outpatient diseases such as acute otitis media or sinusitis, which accounted for 75% of total pneumococcal disease, only accounted for 16% of direct medical costs. With regard to outpatient medication, we only considered antibiotic treatment as it must be dispensed under prescription (unlike other treatments such as antipyretics or anti-inflammatory drugs) and therefore its use could be associated with IPD. However, the costs of outpatient medication are very low compared with the total direct medical costs. Likewise, the effect of the PCV10 on the additional serotypes 1, 5, and 7F was not studied. However, in our setting, PCV10 coverage was approximately 4%, compared with 63.3% for the PCV13 [35,45]. Another limitation is that the results on the costs of IPD must be framed within our geographical area, since these are conditioned by our health system, health policies, circulating serotypes, and number of cases. However, this study may help to better understand the evolution of these costs.

## 5. Conclusions

Following the introduction of the PCV13, the direct costs associated with IPD in children aged < 5 years in Catalonia (Spain) decreased globally and for all clinical presentations due to a decrease in the number of cases. IPD cases during the PCV13 period were more severe, consumed more health resources, and had a higher mean cost. There was a significant decrease in the direct cost associated with IPD cases produced by PCV7 serotypes and the serotypes added to the PCV13, but not in non-PCV13 serotypes. The increase in costs during 2015 could be explained by the increase in the costs associated with non-PCV13 serotypes and serotype 3.

## Figures and Tables

**Table 1:** IPD patient characteristics and health resource use in the two study periods.

Variable	PCV7 Period ^1^ (319 Cases)	PCV13 Period ^2^ (154 Cases)	*p* Value
Sex			
Female	148 (46.4%)	58 (37.7%)	0.073
Male	171 (53.6%)	96 (62.3%)	
**Mean age (SD)**	29.6 (15.7)	26.7 (16.0)	0.057
**Clinical presentation**			
Meningitis	29 (9.1%)	18 (11.7%)	0.273
Septic shock	3 (0.3%)	4 (2.6%)	0.222
Pneumonia	254 (79.6%)	104 (67.5%)	0.004
Non-focal bacteremia	25 (7.8%)	16 (10.4%)	0.355
Osteoarticular infection	6 (1.9%)	7 (4.5%)	0.097
Mastoiditis	-	5 (3.2%)	-
Orbital cellulitis	2 (0.6%)	-	-
**ICU admission**	44 (13.8%)	37 (24.0%)	0.006
**Median ICU stay (SD)**	0.8 (3.1)	1.6 (5.5)	0.086
**Hospital transfer**	77 (24.1%)	52 (33.8%)	0.028
Urgent transfer	11 (3.4%)	20 (13.0%)	>0.001
Non-urgent transfer	66 (20.7%)	32 (20.8%)	0.982
**Mean days of previous hospitalization (SD)**	0.8 (2.0)	0.7 (2.2)	0.587
**Mean days of high-tech hospital hospitalization (SD)**	9.8 (6.3)	10.6 (7.5)	0.226
**Outpatient treatment**	265 (83.1%)	124 (80.5%)	0.496
**Follow up visits**			0.263
Primary care	72 (22.6%)	42 (27.3%)	
Specialist	247 (77.4%)	112 (72.7%)	

IPD, Invasive pneumococcal disease; ^1^ 2007–2009; ^2^ 2012–2015.

**Table 2:** Annual evolution of costs associated with IPD according to health resources.

Year (Cases)	2007 (88)	2008 (98)	2009 (133)	2012 (45)	2013 (34)	2014 (35)	2015 (40)
Days of previous hospitalization	€16,750.4	€12,723.8	€11,596.4	€5959.3	€4026.5	€805.3	€6281.4
ICU stay	€58,500.0	€67,600.0	€209,300.0	€75,400.0	€93,600.0	€35,100.0	€122,200.0
Days of high technology hospital hospitalization	€160,566.3	€176,936.4	€207,238.5	€80,109.0	€87,597.5	€54,334.8	€62,345.7
Days of outpatient antibiotics	€493.1	€621.5	€809.3	€328.7	€171.9	€190.2	€218.6
Urgent hospital transfer	€327.0	€490.6	€981.1	€654.1	€981.1	€817.6	€817.6
Non-urgent hospital transfer	€1635.2	€1880.5	€1880.5	€899.4	€735.8	€327.0	€654.1
Hospital follow-up visits	€7830.0	€10,461.3	€11,359.9	€4043.3	€3209.0	€2374.7	€3979.2
Primary care follow-up visits	€660.0	€390.0	€1110.0	€330.0	€270.0	€420.0	€240.0
Total	€246,761.9	€271,104.2	€444,275.6	€167,723.8	€190,591.8	€94,369.6	€196,736.5
Total per patient	€2804.1	€2766.4	€3340.4	€3728.0	€5605.6	€2696.3	€4981.4

IPD, Invasive pneumococcal disease; all costs in 2014 euro.

**Table 3:** Variation in the mean cost of health resources associated with IPD according to clinical presentation and study period.

Health Resources	Meningitis			Pneumonia			Non-Focal Bacteremia			Other Focal IPD			Total		
	PCV7 Period	PCV13 Period	Percent	PCV7 Period	PCV13 Period	%	PCV7 Period	PCV13 Period	Percent	PCV7 Period	PCV13 Period	Percent	PCV7 Period	PCV13 Period	Percent
Days of previous hospitalization (SD)	€61.1 (174.4)	€268.4 (665.2)	339.4%	€154.7 (356.9)	€116.2 (312.9)	−24.9%	€0.0 (0.0)	€0.0 (0.0)	-	€0.0 (0.0)	€10.1 (40.3)	-	€128.7 (329.5)	€110.9 (347.2)	−13.9%
ICU stay (SD)	€5872.4 (7766.0)	€5272.2 (4356.8)	−10.2%	€501.6 (2915.1)	€1700.0 (8335.9)	238.9%	€0.0 (0.0)	€325.0 (1300.0)	-	€3427.3 (7674.9)	€650.0 (1424.1)	−81.0%	€1051.4 (4070.2)	€1865.6 (7140.4)	77.4%
Days of high-tech hospital hospitalization (SD)	€2474.1 (1871.2)	€3096.0 (1882.8)	25.1%	€1699.7 (976.8)	€1686.2 (935.9)	−0.8%	€1037.9 (644.7)	€1327.9 (1656.9)	27.9%	€1393.2 (678.9)	€2002.7 (1500.8)	43.7%	€1707.6 (1098.0)	€1846.7 (1305.9)	8.1%
Days of outpatient antibiotics (SD)	€0.7 (2.3)	€0.7 (2.4)	3.1%	€6.6 (3.8)	€6.5 (4.9)	−0.5%	€4.9 (3.9)	€4.9 (2.7)	−0.7%	€9.8 (9.8)	€10.1 (6.7)	3.4%	€6.0 (4.4)	€5.9 (5.3)	0.6%
Urgent hospital transfer (SD)	€39.5 (71.2)	€81.8 (84.1)	107.1%	€1.9 (17.7)	€12.6 (43.8)	551.3%	€0.0 (0.0)	€0.0 (0.0)	-	€14.9 (49.3)	€30.7 (65.9)	106.2%	€5.6 (29.9)	€21.2 (55.1)	276.6%
Non-urgent hospital transfer (SD)	€0.0 (0.0)	€4.5 (19.3)	-	€20.9 (35.7)	€22.0 (36.4)	5.2%	€3.3 (16.3)	€5.1 (20.4)	56.2%	€0.0 (0.0)	€10.2 (27.9)	-	€16.9 (33.2)	€17.0 (33.3)	0.4%
Hospital follow-up visits (SD)	€128.4 (0.0)	€128.4 (0.0)	0.0%	€96.5 (50.6)	€88.9 (55.3)	−7.9%	€0.0 (0.0)	€0.0 (0.0)	-	€128.4 (0.0)	€128.4 (0.0)	0.0%	€92.9 (53.7)	€88.3 (56.9)	−4.9%
Primary care follow-up visits (SD)	€0.00 (0.0)	€0.0 (0.0)	-	€5.6 (11.7)	€7.5 (13.0)	35.1%	€30.0 (0.0)	€30.0 (0.0)	0.0%	€0.0 (0.0)	€0.0 (0.0)	-	€6.8 (12.6)	€8.2 (13.4)	20.8%
Total (SD)	€8576.5 (9114,1)	€8852.0 (5135,7)	3.2%	€2487.5 (3118.6)	€3639.9 (8522.3)	46.3%	€1076.1 (648.9)	€1692.9 (2043.7)	57.3%	€4973.5 (7540.3)	€2842.1 (2256.6)	−42.8%	€3016.1 (4510.2)	€3963.9 (7493.6)	31.4%

IPD, Invasive pneumococcal disease; PCV7 period: 2007–2009; PCV13 period: 2012–2015.

## References

[1] O’Brien K.L., Wolfson L.J., Watt J.P., Henkle E., Deloria Knoll M., McCall N., Lee E., Mulholland K., Levine O.S., Cherian T. (2009). Burden of disease caused by *Streptococcus pneumoniae* in children younger than 5 years: Global estimates. Lancet.

[2] Wahl B., O’Brien K.L., Greenbaum A., Majumder A., Liu L., Chu Y., Lukšić I., Nair H., McAllister D.A., Campbell H. (2018). Burden of *Streptococcus pneumoniae* and *Haemophilus influenzae* type b disease in children in the era of conjugate vaccines: Global, regional, and national estimates for 2000–15. Lancet Glob. Health.

[3] World Health Organization (2019). Pneumococcal conjugate vaccines in infants and children under 5 years of age: WHO position paper—February 2019. Wkly. Epidemiol. Rec..

[4] Summary of Prevenar 7 Product Characteristics. European Medicines Agency. http://www.ema.europa.eu/docs/en_GB/document_library/EPAR_-_Summary_for_the_public/human/000323/WC500041558.pdf.

[5] Summary of Synflorix Product Characteristics. European Medicines Agency. http://www.ema.europa.eu/docs/en_GB/document_library/EPAR_-_Pro-duct_Information/human/000973/WC500054346.pdf.

[6] Summary of Prevenar 13 Product Characteristics. European Medicines Agency. https://www.ema.europa.eu/en/documents/overview/prevenar-epar-summary-public_en.pdf.

[7] European Centre for Disease Prevention and Control (2019). Invasive Pneumococcal Disease.

[8] Ben-Shimol S., Greenberg D., Givon-Lavi N., Schlesinger Y., Somekh E., Aviner S., Miron D., Dagan R. (2014). Early impact of sequential introduction of 7-valent and 13-valent pneumococcal conjugate vaccine on IPD in Israeli children <5 years: An active prospective nationwide surveillance. Vaccine.

[9] Fortunato F., Martinelli D., Cappelli M.G., Cozza V., Prato R. (2015). Impact of pneumococcal conjugate universal routine vaccination on pneumococcal disease in Italian children. J. Immunol. Res..

[10] Griffin M.R., Mitchel E., Moore M.R., Whitney C.G., Grijalva C.G. (2014). Declines in pneumonia hospitalizations of children aged <2 years associated with the use of pneumococcal conjugate vaccines—Tennessee, 1998–2012. MWR Morb. Mortal. Wkly Rep..

[11] Lepoutre A., Varon E., Georges S., Dorléans F., Janoir C., Gutmann L., Lévy-Bruhl D. (2015). Impact of the pneumococcal conjugate vaccines on invasive pneumococcal disease in France, 2001-2012. Vaccine.

[12] Guevara M., Ezpeleta C., Gil-Setas A., Torroba L., Beristain X., Aguinaga A., García-Irure J.J., Navascués A., García-Cenoz M., Castilla J. (2014). Reduced incidence of invasive pneumococcal disease after introduction of the 13-valent conjugate vaccine in Navarre, Spain, 2001-2013. Vaccine.

[13] Moore M.R., Link-Gelles R., Schaffner W., Lynfield R., Lexau C., Bennett N.M., Petit S., Zansky S.M., Harrison L.H., Reingold A. (2015). Effect of use of 13-valent pneumococcal conjugate vaccine in children on invasive pneumococcal disease in children and adults in the USA: Analysis of multisite, population-based surveillance. Lancet Infect. Dis..

[14] Picazo J., Ruiz-Contreras J., Casado-Flores J., Negreira S., García-de-Miguel M.J., Hernández-Sampelayo T., Otheo E., Méndez C. (2013). Expansion of serotype coverage in the universal pediatric vaccination calendar: Short-term effects on age- and serotype-dependent incidence of invasive pneumococcal clinical presentations in Madrid, Spain. Clin. Vaccine Immunol..

[15] Picazo J., Ruiz-Contreras J., Casado-Flores J., Giangaspro E., García-de-Miguel M.J., Hernández-Sampelayo T., Otheo E., Méndez C. (2013). Impact of introduction of conjugate vaccines in the vaccination schedule on the incidence of pediatric invasive pneumococcal disease requiring hospitalization in Madrid 2007 to 2011. Pediatr. Infect Dis. J..

[16] Waight P.A., Andrews N.J., Ladhani N.J., Sheppard C.L., Slack M.P., Miller E. (2015). Effect of the 13-valent pneumococcal conjugate vaccine on invasive pneumococcal disease in England and Wales 4 years after its introduction: An observational cohort study. Lancet Infect. Dis..

[17] van de Vooren K., Duranti S., Curto A., Garattini L. (2014). Cost effectiveness of the new pneumococcal vaccines: A systematic review of European studies. Pharmacoeconomics.

[18] Zhang S., Sammon P.M., King I., Andrade A.L., Toscano C.M., Araujo S.N., Sinha A., Madhi S.A., Khandaker G., Yin J.K. (2016). Cost of management of severe pneumonia in young children: Systematic analysis. J. Glob. Health.

[19] Brotons P., Gelabert G., Launes C., Sicuri E., Pallares R., Muñoz-Almagro C. (2013). Cost of hospitalizing children with invasive pneumococcal pneumonia. Vaccine.

[20] Baldo V., Cocchio S., Baldovin T., Buja A., Furlan P., Bertoncello C., Russo F., Saia M. (2014). A population-based study on the impact of hospitalization for pneumonia in different age groups. BMC Infect Dis..

[21] Spoorenberg S.M., Bos W.J., Heijligenberg R., Voorn P.G., Grutters J.C., Rijkers G.T., van de Garde E.M. (2014). Microbial aetiology, outcomes, and costs of hospitalisation for community-acquired pneumonia; an observational analysis. BMC Infect. Dis..

[22] Song J.Y., Choi J.Y., Lee J.S., Bae I.G., Kim Y.K., Sohn J.W., Jo Y.M., Choi W.S., Lee J., Park K.H. (2013). Clinical and economic burden of invasive pneumococcal disease in adults: A multicenter hospital-based study. BMC Infect Dis..

[23] Calderón C., Dennis R. (2014). Economic cost of *Streptococcus pneumoniae* community-acquired pneumonia, meningitis and bacteremia in an adult population that required hospitalization in Bogotá, Colombia. Biomedica.

[24] Registre del Conjunt Mínim Bàsic de Dades (CMBD) dels Hospital D’aguts (2016). Servei Català de la Salut. http://catsalut.gencat.cat/ca/proveidors-professionals/registres-catalegs/registres/cmbd/informes-anuals/.

[25] Tarragó D., Fenoll A., Sánchez-Tatay D., Arroyo L.A., Muñoz-Almagro C., Esteva C., Hausdorff W.P., Casal J., Obando I. (2008). Identification of pneumococcal serotypes from culture-negative clinical specimens by novel real-time PCR. Clin. Microbiol. Infect..

[26] World Health Organization (2019). Neisseria Meningitidis, Streptococcus Pneumoniae, Diagnosis of Meningitis Caused by Laboratory Methods for the and Haemophilus Influenzae: WHO Manual.

[27] Fenoll A., Jado I., Vicioso D., Casal J. (1997). Dot blot assay for the serotyping of pneumococci. J. Clin. Microbiol..

[28] Selva L., Berger C., Garcia-Garcia J.J., de Paz H., Nadal D., Muñoz-Almagro C. (2014). Direct identification of *Streptococcus pneumoniae* capsular types in pleural fluids by using multiplex PCR combined with automated fluorescence-based capillary electrophoresis. J. Clin. Microbiol..

[29] DECRET 170/2010, de 16 de Novembre, de Regulació del Sistema de Pagament dels Convenis i Contractes de Gestió de Serveis Assistencials en l’àmbit del Servei Català de la Salut. https://portaljuridic.gencat.cat/eli/es-ct/d/2010/11/16/170.

[30] ORDRE SLT/79/2014, de 12 de Març, per la Qual es Determinen per a L’any 2014 els Preus Unitaris i la Resta de Valors a Què es Refereix L’article 5 del Decret 170/2010, de 16 de Novembre, de Regulació del Sistema de Pagament de Serveis Sanitaris en L’àmbit del Servei Català de la Salut. https://portaljuridic.gencat.cat/eli/es-ct/o/2014/03/12/slt79.

[31] Navas E., Torner N., Broner S., Godoy P., Martínez A., Bartolomé R., Domínguez A., Working Group for the Study of Outbreaks of Acute Gastroenteritis in Catalonia (Spain) (2015). Economic costs of outbreaks of acute viral gastroenteritis due to norovirus in Catalonia (Spain), 2010–2011. BMC Public Health.

[32] Ordre SLT/99/2013, de 24 de Maig, per la Qual S’estableixen per a L’any 2013 les Tarifes Màximes dels Serveis de Transport Sanitari no Urgent que Convingui o Contracti el Servei Català de la Salut. https://portaljuridic.gencat.cat/eli/es-ct/o/2013/05/24/slt99.

[33] ORDRE SLT/78/2014, de 12 de Març, per la qual es Prorroguen per a L’any 2014 les Tarifes Màximes Corresponents a la Prestació i Concertació de Determinats Serveis Sanitaris. https://portaljuridic.gencat.cat/eli/es-ct/o/2014/03/12/slt78.

[34] Ceyhan M., Ozsurekci Y., Aykac K., Hacibedel B., Ozbilgili E. (2018). Economic burden of pneumococcal infections in children under 5 years of age. Hum. Vaccin Immunother..

[35] Hernández S., Muñoz-Almagro C., Ciruela P., Soldevila N., Izquierdo C., Codina M.G., Díaz A., Moraga-Llop F., García-García J.J., Domínguez Á. (2019). Invasive pneumococcal disease and influenza activity in a pediatric population: Impact of PCV13 vaccination in pandemic and nonpandemic influenza periods. J. Clin. Microbiol..

[36] Lagos R., Muñoz A., Espinoza A., Dowes A., Ruttimann R., Colindres R., Levine M.M. (2009). Direct medical costs of invasive pneumococcal disease and radiologically-diagnosed pneumonia among Chilean children. Rev. Panam Salud Pública.

[37] Ricketson L.J., Conradi N.G., Vanderkooi O.G., Kellner J.D. (2018). Changes in the nature and severity of invasive pneumococcal disease in children before and after the seven-valent and thirteen-valent pneumococcal conjugate vaccine programs in Calgary, Canada. Pediatr. Infect Dis. J..

[38] Ciruela P., Izquierdo C., Broner S., Muñoz-Almagro C., Hernández S., Ardanuy C., Pallarés R., Domínguez A., Jané M., Catalan Working Group on Invasive Pneumococcal Disease (2018). The changing epidemiology of invasive pneumococcal disease after PCV13 vaccination in a country with intermediate vaccination coverage. Vaccine.

[39] Muñoz-Almagro C., Jordan I., Gene A., Latorre C., Garcia-Garcia J.J., Pallares R. (2008). Emergence of invasive pneumococcal disease caused by nonvaccine serotypes in the era of 7-valent conjugate vaccine. Clin. Infect. Dis..

[40] Feikin D.R., Kagucia E.W., Loo J.D., Link-Gelles R., Puhan M.A., Cherian T., Levine O.S., Whitney C.G., O’Brien K.L., Moore M.R. (2013). Serotype-specific changes in invasive pneumococcal disease after pneumococcal conjugate vaccine introduction: A pooled analysis of multiple surveillance sites. PLoS Med..

[41] Goettler D., Streng A., Kemmling D., Schoen C., von Kries R., Rose M.A., van der Linden MLiese J.G. (2020). Increase in *Streptococcus pneumoniae* serotype 3 associated parapneumonic pleural effusion/empyema after the introduction of PCV13 in Germany. Vaccine.

[42] Domínguez Á., Ciruela P., Hernández S., García-García J.J., Soldevila N., Izquierdo C., Moraga-Llop F., Díaz A., F de Sevilla M., González-Peris S. (2017). Effectiveness of the 13-valent pneumococcal conjugate vaccine in preventing invasive pneumococcal disease in children aged 7-59 months. A matched case-control study. PLoS ONE.

[43] Hernández S., Moraga-Llop F., Díaz A., de Sevilla M.F., Ciruela P., Muñoz-Almagro C., Codina G., Campins M., García-García J.J., Esteva C. (2020). Failures of 13-valent conjugated pneumococcal vaccine in age-appropriately vaccinated children 2–59 months of age, Spain. Emerg. Infect. Dis..

[44] Huang S.S., Johnson K.M., Ray G.T., Wroe P., Lieu T.A., Moore M.R., Zell E.R., Linder J.A., Grijalva C.G., Metlay J.P. (2011). Healthcare utilization and cost of pneumococcal disease in the United States. Vaccine.

[45] Savulescu C., Krizova P., Lepoutre A., Mereckiene J., Vestrheim D.F., Ciruela P., Ordobas M., Guevara M., McDonald E., Morfeldt E. (2017). Effect of high-valency pneumococcal conjugate vaccines on invasive pneumococcal disease in children in SpIDnet countries: An observational multicentre study. Lancet Respir. Med..

